# The interface between spoken and written language: developmental disorders

**DOI:** 10.1098/rstb.2012.0395

**Published:** 2014-01-19

**Authors:** Charles Hulme, Margaret J. Snowling

**Affiliations:** 1 Division of Psychology and Language Science, University College London, 2 Wakefield Street, London WC1N 1PF, UK; 2 St John's College, St Giles', Oxford OX1 3JP, UK

**Keywords:** reading, decoding, dyslexia, reading comprehension impairment, intervention, causes

## Abstract

We review current knowledge about reading development and the origins of difficulties in learning to read. We distinguish between the processes involved in learning to decode print, and the processes involved in reading for meaning (reading comprehension). At a cognitive level, difficulties in learning to read appear to be predominantly caused by deficits in underlying oral language skills. The development of decoding skills appears to depend critically upon phonological language skills, and variations in phoneme awareness, letter–sound knowledge and rapid automatized naming each appear to be causally related to problems in learning to read. Reading comprehension difficulties in contrast appear to be critically dependent on a range of oral language comprehension skills (including vocabulary knowledge and grammatical, morphological and pragmatic skills).

## 1. Introduction

In this review, we will consider a wide range of evidence about the inter-relationships between developmental disorders of reading and spoken language. When talking about spoken and written language development and their disorders, it is important to distinguish between the different component skills of both. For spoken language, it is common to distinguish between four domains: phonology, grammar, semantics and pragmatics. When we consider written language, we need to distinguish between reading and spelling. Within the domain of reading, it is important to make a further distinction between decoding (usually assessed by the accuracy or fluency of reading aloud) and comprehension (the adequacy of understanding text, usually assessed by questions about the meaning of a passage).

Mattingly [1, p. 133] famously proposed that ‘reading is parasitic on speech’. He was correct in the sense that a child's ability to learn to recode print (which was the topic of his chapter) is critically dependent on their phonological, or speech sound, skills. If, however, we accept that reading comprehension depends on both decoding and language comprehension skills (Gough & Tunmer's [2] simple view of reading), there is no doubt that broader oral language skills (including grammar, semantics and pragmatics) are also important for reading comprehension. In short, reading for meaning depends on all four domains of oral language.

The intimate relationship between spoken and written language skills has been long accepted in studies of development, but perhaps less so in studies of acquired disorders of these skills. However, the primary systems hypothesis [3,4] sees adult cases of reading disorders, just like developmental cases, as reflecting impairments to underlying primary brain systems (systems concerned with different aspects of oral language as well as visual processing mechanisms). This view suggests that models of acquired and developmental disorders of reading already show a good degree of alignment.

## 2. Models of reading development and reading disorders

Understanding a disorder of development depends on having a model of normal development for the skill in question [5]. More broadly, any complete model of cognitive performance in adulthood needs to be compatible with evidence about how the process in question developed. Studies of development can, in turn, inform and constrain theories of adult psychological functioning. To quote Baldwin [6, p. 5], ‘the study of children is often the only way we have of testing our mental analyses. If we decide that a certain complex product is due to the union of simple mental elements, then we may appeal to the proper period of child life to see the union taking place’.

The argument that is at the heart of this review, that language problems are the predominant causes of problems in learning to read, rests upon two different models of how language processes operate to determine the course of normal reading development: the triangle model of word reading and Gough & Tunmer's [2] simple view of reading.

Currently, there are two influential classes of model of adult word reading: dual-route [7] and connectionist models. Dual-route models conceptualize adult word recognition as depending upon independent lexical and sublexical routes from the written form of a word to its pronunciation. These theories are essentially ‘static’ models of adult performance and do not provide any account of how the systems they postulate develop. By contrast, connectionist theories of word reading are explicitly developmental and see word reading as being dependent upon the integrity of phonological and semantic representations that exist in the language processing system before reading develops. Among connectionist theories, the main variant is often referred to as the ‘triangle’ model [8] and provides an extremely productive framework for thinking about disorders of reading development.

According to the triangle model, learning to read essentially consists of creating mappings or associations between visual representations of the letter strings that constitute words (orthographic representations) and the phonological and semantic representations of spoken language that correspond to those words. As a result of training (simulating learning to read), such a model develops two interacting ‘pathways’ that work together to read individual words. The ‘phonological pathway’ maps orthography onto phonology: that is, given a written word as input, it can be translated into its corresponding spoken form. The ‘semantic pathway’ maps orthography onto phonology via semantics: a written word as input produces direct activation of the word's meaning, which in turn activates pronunciation.

An assumption of one variant of the triangle model [9] is that at the beginning of reading development, the child's cognitive resources are devoted to establishing the ‘phonological pathway’ (the system for mapping letters on to sounds) but that later, reading comes to rely increasingly on the ‘semantic pathway’. The use of the semantic pathway may be particularly important for the reading of exception words that the phonological pathway does not handle efficiently. Indeed, as training of the model proceeds, there is a ‘division of labour’ such that the semantic pathway begins to favour exception word reading, whereas the phonological pathway becomes specialized for reading novel words that the system has not encountered before.

As noted earlier, it is important to distinguish between the ability to read words accurately and fluently and the ability to comprehend text. Accurate and fluent word reading are essential for good reading comprehension. Gough & Tunmer's [2] simple view of reading underlines the fact that reading comprehension is the product of both decoding skill and oral language comprehension (reading comprehension = decoding × listening comprehension). It follows from this model that problems with reading comprehension can arise from two different sources (problems with decoding or problems with oral language comprehension). Children with decoding problems are usually referred to as having developmental dyslexia. Children with adequate decoding but problems purely with reading comprehension are usually referred to as having reading comprehension impairment (or more simply as ‘poor comprehenders’). The existence of both of these groups of children, who will be discussed below, is exactly what we would expect from the simple view of reading.

In summary, both the triangle model and the simple view of reading, see the development of reading skills as ‘parasitic’, not specifically on ‘speech’ but rather on earlier developing oral language skills. This will be the main focus of this review.

## 3. Developing and testing causal theories of developmental disorders

The issue that lies at the heart of developmental psychology is an attempt to establish the causes of development. The idea that learning to read is parasitic on earlier developing oral language skills is a broad and non-specific causal theory. In the sections that follow, this general theory will be fleshed out. Before doing so, it is useful to reflect on the sorts of evidence we can use to test causal theories in this area.

Ultimately, all developmental disorders can be conceptualized as the product of interactions between genetic and environmental risk factors [5]. For present purposes however, we will focus on a cognitive level of explanation that links brain mechanisms to behaviour. In relation to reading disorders, this approach essentially focuses on trying to establish causal links between deficits in specific aspects of oral language skills and aspects of reading development. The approaches that have been developed to evaluate putative causal relationships involve a number of steps. Any hypothetical cause must exist prior to its proposed consequence (the ‘logic of causal order’; see [10]). Establishing that variations in a given oral language skill (e.g. phoneme awareness, PA) that exist prior to learning to read are strong correlates of later variations in word reading skill suggests, but does not establish, that a causal effect exists. In some cases where training studies are not practicable or ethical our only way of testing causal theories may be to conduct longitudinal studies and evaluate alternative interpretations for putative causal links. The approach, essentially, is to show that the relationship between a potential cause (e.g. phonemic awareness) and its consequence (word reading skill) cannot be explained by other confounding variables (a child's IQ, or their educational background, for example).

Ultimately, however, to provide convincing evidence for causal hypotheses, we need to conduct training studies. If we can show in an experiment that training a particular oral language skill (e.g. phonemic awareness) leads to improvements in later reading skills, then we have much better evidence of a causal relationship. Finally, if we can measure the functioning of a hypothetical mechanism (levels of phonemic awareness) that is believed to be responsible for producing improvements in reading outcomes, then we can assess the extent to which changes in an outcome (reading) are directly proportional to changes in the intervening mechanism (PA) in a mediation analyses (see [11]). Put simply, if an intervention produces effects via an intermediate mechanism, then variations in the effectiveness of the intervention across individuals should be proportional to variations in the changes brought about in the hypothetical mechanism (if reading improves because PA has improved, then improvements in reading should vary across individuals in line with improvements in PA).

## 4. Possible causal relationships between impairments of spoken and written language

### (a) Disorders of reading accuracy and fluency

A necessary step towards becoming a skilled reader is the acquisition of efficient decoding skills: an accomplishment that represents a significant obstacle for children with dyslexia. However, because reading skills, including decoding abilities, show continuous variation within the population, where the cut-off for dyslexia is set is to some extent arbitrary; current estimates suggest that somewhere between 3 and 7% of the population experience educationally significant difficulties in this area (see [5,12], for further details).

If we accept that dyslexia represents the lower end of a continuous distribution of decoding skills in the population, then to explain dyslexia, we need to understand the cognitive mechanisms that are causally linked to variations in decoding skills. There is now good evidence that there are three main predictors of individual differences in the early stages of learning to decode in alphabetic languages: letter knowledge (LK), PA and rapid automatized naming (RAN) [13–15]. Arguably, most research has sought to understand the role of PA and whether it is a cause or a consequence of learning to read [16,17]. Current evidence is consistent with the notion that variations in PA, and letter–sound knowledge, are two factors that have a causal influence on the development of decoding. RAN appears likely to be another causal influence on decoding skill although here the evidence for causation is more equivocal. Evidence from studies of children at familial risk of dyslexia indicates that early in development children who go on to develop dyslexia have relatively broad oral language weaknesses that affect vocabulary knowledge and naming skills as well as phonological oral language skills [18].

#### (i) Phoneme awareness

Measures of PA involve children manipulating or making judgements about the phonemic units in spoken words. Many concurrent and longitudinal studies have assessed the relationship between PA and children's reading ability. In a meta-analysis of these studies, which included both extreme group comparisons (comparing dyslexic with typically developing children) and correlational studies of unselected samples [19], children with dyslexia showed a large deficit in PA in comparison with typically developing children of the same age (pooled effect size estimate *d* = −1.37) and younger children matched on reading level (pooled effect size estimate *d* = −0.57). Analyses of studies of unselected samples showed that phonemic awareness was a strong correlate of individual differences in word reading ability, and that this effect remained reliable after controlling for variations in both verbal short-term memory and awareness of the onset-rime components of words.

#### (ii) Letter knowledge

LK is also a predictor of variations in children's word reading ability. Moderate correlations have been reported between LK assessed at the start of formal reading instruction and word reading skills measured later that year or early the next year [14,20,21]. In different studies, LK has been assessed using measures of either letter–sound knowledge, letter–name knowledge or both. These two measures are typically moderately correlated with each other. Theoretically, however, it is letter–sound knowledge which is likely to be a critical determinant of variations in children's ability to learn to read, because it is one of the foundations of the alphabetic principle [22].

#### (iii) Phoneme awareness and letter knowledge: issues of causation

There are close associations between PA and LK and learning to read. Both these effects operate longitudinally from an age when reading skills are very limited [14,21] suggesting that they may reflect causal influences on learning to read. Direct evidence for causation requires training studies. There is evidence that training phonemic awareness in children is effective in helping to improve word reading skills, especially when such training is coupled with appropriate phonically based reading instruction. For example, a meta-analysis [23] reported an effect size of *d* = 0.67 (based on seven studies) for training phonemic awareness on word reading.

**Figure 1.**
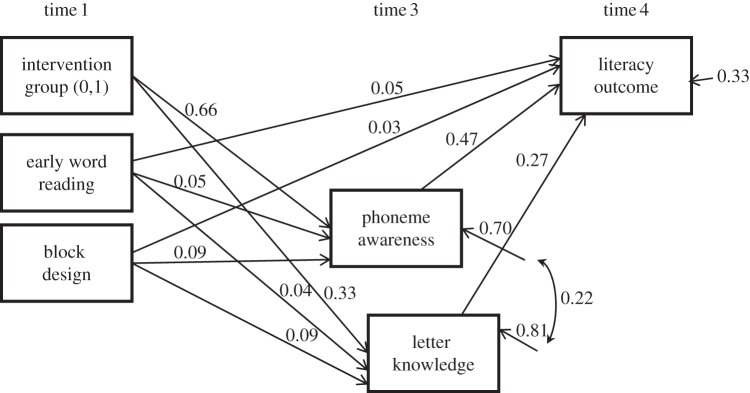
Path model showing that the effects of a phonology + reading intervention are mediated by its effects on phoneme awareness and letter knowledge. (Adapted from Hulme *et al*. [25].)

A study by Bowyer-Crane *et al*. [24] provides further evidence for the causal role of PA and letter–sound knowledge in learning to read. This study delivered a phonology with reading intervention programme (which trained letter–sound knowledge and PA alongside book work) to young children selected for having weak oral language skills at school entry. This ‘phonology with reading’ intervention produced significant improvements (in comparison with a control group who were given an oral language intervention) in later word-level reading and spelling skills. A re-analysis of data from this study [25] showed that improvements in letter–sound knowledge and PA measured at the end of the intervention fully accounted for (mediated) the improvements seen in the children's reading and spelling skills measured five months after the intervention had finished (figure 1).

Because this study randomly assigned children to two different interventions (a phonology and reading versus an oral language intervention programme), we have good evidence that the improvements seen in letter–sound knowledge, PA and literacy skills are causal effects. However, perhaps more critically, the results of the mediation model provide support for the theory that motivated the intervention. The mediation model provides evidence that weaknesses in two underlying skills (PA and letter–sound knowledge) are two causes of difficulties in learning to read. An intervention that targeted these two skills was effective in improving reading, with the improvements observed in reading being proportional to the improvements seen in these two underlying skills.

#### (iv) Rapid automatized naming

One other important predictor of variations in reading development is RAN. RAN tasks require a child to name as quickly as they can a list of pictures, colours, letters or numbers. Children with dyslexia perform poorly on RAN [26], and in unselected samples of children, there are reliable concurrent and longitudinal correlations between RAN and children's reading skills (see [27,28], for reviews). The fact that even RAN for pictures and colours, measured before children can read, is predictive of later variations in reading skills [21] indicates that this effect cannot just be a consequence of differences in LK. The fact that RAN predicts reading accuracy as well as fluency suggest that this effect does not simply reflect general variations in speed of processing. Furthermore, whatever RAN taps, it is statistically independent of LK and PA as a predictor of decoding skill. However, we would emphasize that there is no evidence from a training study to clinch the argument that RAN plays a causal role in constraining the rate of reading development; on the contrary, training in rapid letter naming appears to affect neither RAN nor reading reliably [28].

## 5. The role of rapid automatized naming and letter knowledge as putative causal influences on the development of word decoding

At the moment, the relationship between RAN and LK and their possible causal roles in learning to read is a little uncertain. It has been argued [21] that RAN is an index of the efficiency of a left-hemisphere brain circuit that underlies object naming and that this circuit is recruited to form the basis of the visual word recognition system. However, in cognitive terms, it seems that measures of both LK (giving the sound for a letter in the absence of time pressure) and RAN (saying the names of a list of objects, colours, letters or digits as quickly as possible) must depend upon a form of cross-modal associative learning (associating what is seen with a name). This raises the possibility that the reason both RAN and LK correlate with word reading skill is that they both tap the integrity of a cross-modal visual–verbal associative learning mechanism (and such a mechanism is obviously likely to be critical for learning to read as well).

There is evidence that variations in verbal associative learning are related to variations in learning to read. Hulme *et al*. [29] showed that a measure of visual–verbal paired-associate learning (associating abstract shapes with a nonsense word) was a concurrent predictor of individual differences in word reading skill, which was independent of PA (which was also a predictor of reading skill). In a further study, it was shown [30] that the same measure of visual–verbal paired-associate learning was also a significant concurrent predictor of reading ability in slightly older children, even after controlling for PA and RAN (which were also unique predictors). Moreover, a further striking finding [31] was that the variance in common between two measures of paired-associate learning (visual–verbal and verbal–verbal) was a unique concurrent predictor of reading skill, as were RAN and phoneme deletion ability. In summary, it is clear that verbal paired-associate learning (PAL) ability is a concurrent correlate of reading skills in the early to middle school years. Similarly, studies of children with dyslexia have shown them to have impairments on such paired-associate learning tasks [32,33].

One clear limitation of these studies of PAL, however, is that they all have a concurrent rather than longitudinal design. Lervag *et al*. [30] reported a large-scale (*N* = 234) longitudinal study of Norwegian children starting before formal reading instruction had begun. Strikingly, PA, RAN and LK measured at time 1 (roughly nine months before formal reading instruction began) were significant unique predictors of reading ability assessed at time 2 (roughly three months after formal reading instruction had begun). However, paired-associate learning ability was not a predictor of reading ability here (and nor were measures of verbal short-term memory, verbal ability or non-verbal ability).

The hypothesis explored in this study [30] was that RAN may share variance with visual–verbal PAL because it taps the efficiency of name retrieval; in turn, efficient name retrieval depends on the ability to learn the names of objects and symbols—that is, on successful associative learning (poorer or less complete learning of the association between a visual stimulus and its name would lead to slower name retrieval). In their study, however, RAN correlated only modestly with PAL (0.32), and RAN's predictive relationship with reading remained after controlling for the effects of PAL. Furthermore, PAL was not a significant predictor of reading ability longitudinally even if RAN was excluded as a predictor, suggesting that RAN is tapping a different construct to paired-associate learning. Together, these findings imply that PAL itself does not explain variations in the rate of learning to read within the general population. These findings also suggest that there is something highly specific about the speeded retrieval of the names of visual stimuli that is tapped by RAN, that is a critical aspect of the processes involved in learning to read. In line with this, it was found [21] that the duration of pauses between naming successive items (rather than the duration of articulating the items themselves) was the critical component of RAN that predicted later variations in reading skill.

## 6. Does word reading develop in similar ways in different alphabetic scripts?

English is more inconsistent in its mappings between letters and the sounds in words than other alphabetic orthographies that have been studied. This has led some to argue that the relative importance of variations in letter–sound knowledge, phonemic awareness and RAN as predictors of reading ability would differ in English in comparison with languages whose orthographies have more consistent spelling–sound correspondences. Some have argued [32] that in consistent orthographies, RAN is the predominant predictor of variations in reading ability (which is usually measured by measures of reading fluency), whereas PA and letter–sound knowledge are much less important. A wide range of studies have examined the predictive relationship between RAN and reading in different languages with rather complex and inconsistent results (for reviews see [13,28]). These inconsistencies likely reflect the fact that different studies have used different measures of reading and RAN.

To try to reconcile these apparent inconsistencies a group of us [13] conducted a large-scale longitudinal study of learning to read, using directly comparable measures, in four languages (English, Spanish, Slovak, Czech). The study began just before or soon after the start of formal reading instruction in all languages and assessed the relative importance of PA, letter–sound knowledge, RAN and verbal memory span measured at the beginning of the study as predictors of reading ability some 10 months later. The findings revealed a remarkably clear pattern with PA, letter–sound knowledge and RAN (but not verbal memory span) being reliable predictors, with equal relative importance, of later reading skills in all four languages. Furthermore, PA was at least as strong a predictor of both reading and spelling development as RAN in this study, even when reading was measured by speeded tests. In sum, this study suggests that the cognitive processes involved in learning to decode print are essentially identical in English and the three other much more consistent European orthographies studied. More generally, there is growing evidence that these three skills might be considered universal markers of the cognitive prerequisites for learning to read across different writing systems—namely symbol knowledge, awareness of the sound units involved in creating mappings between orthography and phonology and RAN [34].

## 7. The role of non-phonological language skills in learning to decode print

The focus so far has been on the role of phonological skills as causal influences on the development of decoding skills. Phonological skills appear to play a dominant role in shaping the early development of decoding skills, but there is evidence that broader oral language skills may also play a role in influencing decoding, particularly in older children.

One such language skill is morphological awareness (awareness of the morphological constituents of spoken words). Several studies have identified morphological awareness as a unique concurrent predictor of variations in word reading ability in older children. For example, one study [35] found that morphological awareness in a sample of 4, 6 and 8 Grade children predicted variations in word reading ability, after controlling for age, phonological awareness and naming speed. There is also evidence that morphological training may be effective in improving word decoding skills. A meta-analysis of training studies [36] showed that interventions, including substantial morphological awareness training, produced moderate improvements (*d* = 0.41) in word reading skills (with evidence of larger effects in less-able readers).

Another oral language skill that seems likely to be related to learning to decode print is vocabulary knowledge. Current evidence, however, suggests that vocabulary knowledge is only weakly related to learning to decode print, at least in the early stages of learning to read. For example, the longitudinal study of Lervag *et al*. [21] found no unique relationship between a latent verbal ability factor (defined by vocabulary and similarities subtests from the Wechsler Preschool and Primary Scale of Intelligence test) and reading ability measured after three months in school. Similarly, a large-scale longitudinal study in England [14] found no unique relationship between receptive vocabulary skills measured at school entry and decoding skills measured after 1 and 2 years of formal instruction (though vocabulary and grammatical skills were unique predictors of reading comprehension skills measured after 2 years in school). It seems likely, however, that vocabulary skills will be more important in older children once they have made progress in understanding the alphabetic principle [37]. There is also evidence from small-scale experimental studies that knowledge of specific word meanings may be important for children learning to read those words [38]. One interpretation of such findings is that word-specific knowledge may be important for learning to read a word (in line with the division of labour idea embodied in the triangle model discussed earlier), but that general measures of the breadth of vocabulary knowledge are relatively insensitive as indices of the word knowledge that are relevant on a given reading test.

## 8. Disorders of reading comprehension

‘Poor comprehenders’ show a marked deficit in reading comprehension in relation to their level of reading accuracy. Given a continuous distribution of reading scores in the population, as in the case of dyslexia, where the cut-off for identifying this type of reading difficulty should be placed is to some extent arbitrary. Hulme & Snowling suggested [39] that children whose reading comprehension standard score is equal to or below 90 and have a reading-accuracy standard score of 90 or above, coupled with a deficit of at least 1 s.d. in reading comprehension standard scores in comparison with reading-accuracy standard scores, should qualify for such ‘diagnosis’. Using this criterion, 3.3% of a nationally representative sample of 1324 children in the UK were identified as poor comprehenders.

According to the simple view of reading [2], reading comprehension (*R*) is equal to decoding (*D*) ‘multiplied by’ linguistic comprehension (*R* = *D* × *C*). Given that the poor comprehender profile is defined by adequate reading accuracy (decoding) coupled with deficient reading comprehension, it follows from the simple view of reading that such children should show deficits on measures of language comprehension. A good deal of evidence supports this idea.

Many studies have shown that poor comprehenders show substantial deficits on a range of measures of oral language comprehension (including measures of vocabulary knowledge, listening comprehension, grammatical and morphological skills) in comparison with age-matched control children ([40–43] for reviews see [5,44]). Furthermore, there is evidence that these language deficits exist prior to learning to read, which supports the idea that they are a plausible cause of these children's reading comprehension difficulties [40,42].

To test the idea that reading comprehension impairment is caused by an underlying weakness in oral language comprehension skills, Hulme and co-workers [45] conducted a randomized trial in which 160 children with poor reading comprehension relative to accuracy were randomly assigned to one of four conditions: oral language training, text comprehension training, combined oral language with text comprehension training or an untreated waiting list control. The interventions were delivered in the children's schools by specially trained teaching assistants in three 30-min sessions each week over 20 weeks. The children's reading and language skills were assessed before the intervention began, after the intervention was completed, and again some 11 months later.

Immediately after the interventions were completed, all three intervention groups showed reliable improvements of statistically equivalent size in reading comprehension (as measured by the Wechsler Individual Achievement test II) in comparison with the control group (increases of approx. 3.5–4.5 standard-score points; effect sizes between *d* = 0.59 and *d* = 0.99). However, at delayed follow-up, 11 months after the intervention had been completed, the advantage of the oral language intervention group had increased to 7.9 standard-score points compared with the untreated control group (*d* = 1.24—a very large effect), and this group was now showing a larger gain than either the text comprehension or the combined text comprehension and oral language groups (gains of 5.2 and 4.7 standard-score points, respectively).

Following the logic described earlier, a mediation analysis was conducted in which changes in vocabulary knowledge measured at the end of the intervention were examined as a possible mediator of the increases in reading comprehension scores at delayed follow-up (some 11 months after the intervention had finished). The effects of both the oral language and the combined oral language and text comprehension interventions were at least partly accounted for by changes in a measure of vocabulary that had been taught in these interventions (the direct effect of the oral language and combined programmes on reading comprehension scores were reduced by roughly 30% by including vocabulary as a mediator of outcome). The children in the oral language intervention (who had experienced a large dose of vocabulary instruction) also showed statistically reliable improvements at the end of the intervention on a standardized test of vocabulary knowledge involving words that had not been taught in the intervention. This, together with the increased size of reading-comprehension advantage at follow-up for this group, suggests that the intervention had resulted in some generalized improvements in these children's oral-language comprehension abilities.

Further support for a causal role of language comprehension skills as determinants of reading comprehension skills comes from another intervention study by our group. That study [46] reported the findings of a broad-based oral language intervention given over 30 weeks to children in nursery and reception classes (when children were from 4 to 5 years old). This intervention was successful in producing reliable improvements in children's oral language and spoken narrative skills in comparison with a waiting list control group. Importantly, the intervention showed generalization to a standardized measure of reading comprehension given to the children at delayed follow-up some six months after the intervention was completed. A mediation analysis showed that the improvements in reading comprehension at delayed follow-up were completely mediated by the improvements seen in a language latent factor (defined by the Clinical Evaluation of Language Fundamentals vocabulary, information and grammar scores from the action picture test and a measure of listening comprehension) assessed immediately after the intervention had been completed.

In summary, the findings of this study and together with those of an earlier study [45] show that weaknesses in oral language comprehension skills can be ameliorated by training, and in line with the simple view of reading, such improvements in oral language skills lead directly to later improvements in children's reading comprehension. The findings of both studies support a causal theory that sees poor reading comprehension as arising from underlying oral language comprehension deficits. More studies are required to identify whether particular subcomponents of language comprehension are particularly critical for the development of children's reading comprehension skills. Some findings [45] indicate that vocabulary knowledge is one skill that contributes to reading comprehension ability, but it seems likely that many other oral language skills are also important (including grammatical and morphological skills). It is also clear that a range of other skills, including non-verbal abilities, appear to predict variations in reading comprehension ability in clinical populations (see [47] for a review) and it remains to determine the extent to which non-linguistic cognitive processes may affect the development of reading comprehension skills. Finally, pragmatic language skills are likely to have effects on the development of reading comprehension. For example, some autistic children, and some non-autistic children who share the pragmatic language difficulties that are common in autism, show problems of reading comprehension that appear to be related to their weak pragmatic language skills (see [48] for a review).

## 9. The origins of language learning weaknesses that underlie reading difficulties

We have focused so far on the proximal causes of developmental disorders of reading, which we have argued reflect a variety of impairments in children's oral language skills. Clearly, a pressing question is to understand the causal risk factors that contribute to these language learning difficulties. Ultimately, variations in oral language skills will reflect differences in genetic and environmental influences in the population studied. Individual differences in both reading and oral language skills appear to be highly heritable.

Twin studies provide evidence for the importance of genetic influences for the genesis of reading problems, as well as for normal variations in reading skills in the population [49]. It has been suggested that as much as 70% of the variation in 7-year-olds’ decoding skills is attributable to genetic differences [50]. Similarly, normal variations in oral language skills are heritable, and there is evidence for the importance of genetic influences in the aetiology of specific language impairment [51,52]. Moreover, recent genetic advances have identified a number of candidate genes associated with dyslexia and specific language impairment [53].

At a cognitive level of explanation, there have been attempts to reduce reading and language disorders to simpler underlying mechanisms. At the time of writing, such attempts have primarily focused on dyslexia. The hypotheses range from extremely broad ideas to more specific proposals. At a broad level, Nicholson & Fawcett [54] suggested that dyslexia might reflect an impairment of the cerebellum which led to an automatization deficit. This theory had difficulties on logical grounds in explaining the highly specific difficulties seen in many children with dyslexia. Subsequent studies suggest that the original evidence suggesting cerebellar impairments in children with dyslexia were actually the result of comorbidity with attention deficit hyperactivity disorder [55,56].

Much more narrowly focused explanations for phonological difficulties have included the idea of them arising from a speech perception [57] or more basic auditory processing [58,59] impairment. However, the evidence for these views is mixed, and there is not yet convincing evidence from longitudinal studies testing causal hypotheses (see [5, ch. 2], for a review). Thus, a question remaining for future research is to find more basic cognitive level explanations for the origin of the language learning weaknesses which appear critical determinants of reading difficulties.

## 10. Summary and conclusion

We have provided a selective review of current knowledge about reading development and the origins of difficulties in learning to read. We have argued that the proximal causes of problems in acquiring adequate decoding and reading comprehension skills are a variety of deficits in underlying oral language skills. We believe our arguments complement well the primary systems hypothesis [3,4], which sees adult cases of reading disorders being heavily influenced by impairments to underlying oral language systems. A pressing need in the developmental field is for more research aimed at understanding the cognitive, neural and genetic mechanisms that contribute to the genesis of language learning difficulties.
